# Supplementary material for: The effects of genital myiasis on the diversity of the vaginal microbiota in female Bactrian camels
Source: BMC Vet Res. 2022 Mar 5;18:87. doi: 10.1186/s12917-022-03189-5 (PMC8897907; doi:10.1186/s12917-022-03189-5)
Supplement: Supplementary file 5 — Additional file 5. [file 12917_2022_3189_MOESM5_ESM.zip › MPL201709200_16s_yy/Treat1/C01_diff/metastats/diff_group_phylum.pdf]

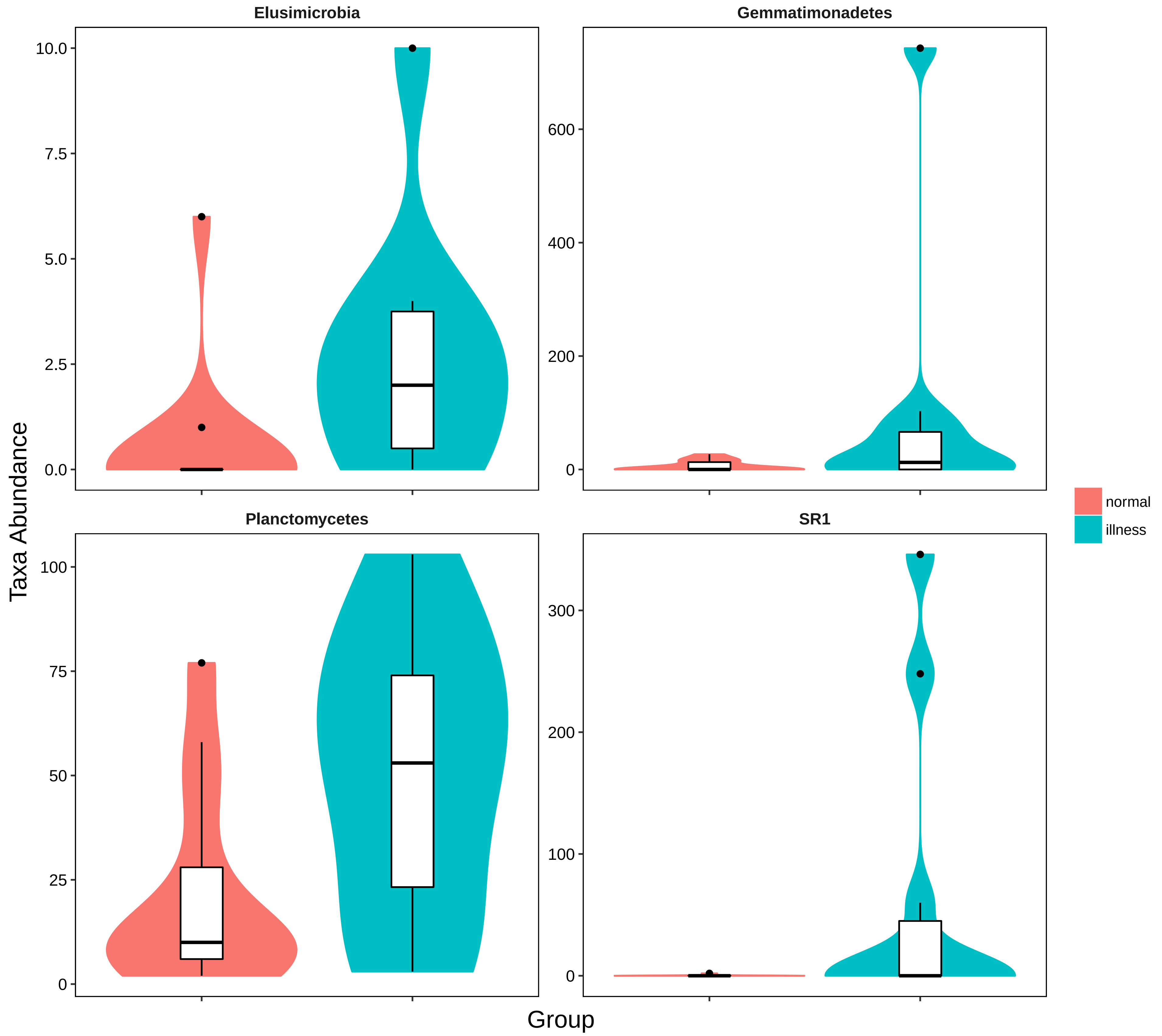
Elusimicrobia
Gemmatimonadetes
Planctomycetes
SR1
10.0
7.5
5.0
2.5
0.0
600
400
200
0
100
75
50
25
0
300
200
100
0
Taxa Abundance
Group
normal
illness
